# Mechanisms of Chronic State of Inflammation as Mediators That Link Obese Adipose Tissue and Metabolic Syndrome

**DOI:** 10.1155/2013/136584

**Published:** 2013-06-13

**Authors:** Eduardo Fuentes, Francisco Fuentes, Gemma Vilahur, Lina Badimon, Iván Palomo

**Affiliations:** ^1^Immunology and Haematology Laboratory, Department of Clinical Biochemistry and Immunohematology, Faculty of Health Sciences, Interdisciplinary Excellence Research Program on Healthy Aging, Universidad de Talca, Talca, Chile; ^2^Centro de Estudios en Alimentos Procesados (CEAP), Conicyt-Regional, Gore Maule, R09I2001 Talca, Chile; ^3^Interno Sexto Año, Escuela de Medicina, Facultad de Medicina, Universidad Católica del Maule, Chile; ^4^Centro de Investigación Cardiovascular, ICCC-CSIC, Hospital de la Santa Creu i Sant Pau, CiberOBN, Instituto Carlos III, Barcelona, Spain

## Abstract

The metabolic syndrome is a cluster of cardiometabolic alterations that include the presence of arterial hypertension, insulin resistance, dyslipidemia, and abdominal obesity. Obesity is associated with a chronic inflammatory response, characterized by abnormal adipokine production, and the activation of proinflammatory signalling pathways resulting in the induction of several biological markers of inflammation. Macrophage and lymphocyte infiltration in adipose tissue may contribute to the pathogenesis of obesity-mediated metabolic disorders. Adiponectin can either act directly on macrophages to shift polarization and/or prime human monocytes into alternative M2-macrophages with anti-inflammatory properties. Meanwhile, the chronic inflammation in adipose tissue is regulated by a series of transcription factors, mainly PPARs and C/EBPs, that in conjunction regulate the expression of hundreds of proteins that participate in the metabolism and storage of lipids and, as such, the secretion by adipocytes. Therefore, the management of the metabolic syndrome requires the development of new therapeutic strategies aimed to alter the main genetic pathways involved in the regulation of adipose tissue metabolism.

## 1. Introduction 

The metabolic syndrome (MS) is a cluster of cardiometabolic alterations that include the presence of arterial hypertension, insulin resistance, dyslipidemia, cardiovascular disease (CVD), and abdominal obesity [1, 2]. MS presents a prothrombotic state as a result of endothelial dysfunction, the presence of a hypercoagulability state produced by an imbalance between coagulation factors and the proteins that regulate fibrinolysis and increased platelet reactivity [3–5]. In this latter regard, we have recently described that obese-diabetic rats with MS have an altered megakaryopoiesis that contributes to increased thrombosis. These alterations are due to an increased platelet turnover caused by a combination of accelerated death and an increased platelet production confirmed by the observation of an increased number of reticulated platelets (the youngest, more immature, and more reactive platelets). Importantly, all these alterations were associated with an increased thrombotic risk, analyzed in vivo by real time intravital microscopy, in wild-type obese-diabetic animals as well as in lean normoglycemic controls transplanted with bone marrow from obese-diabetic donors [6]. Moreover, we have also described that obese nondiabetic rats also show increased platelet counts and an increased mean platelet volume (MPV) which are associated with an increased thrombotic risk (similar to that observed in obese-diabetic rats) [7]. In fact, we have shown that platelet number, MPV, and thrombotic risk are directly correlated with weight and that a reduction of peripheral insulin resistance can contribute to reduce thrombotic risk in obese subjects. These alterations might be a consequence of the low-grade chronic inflammatory state observed in obesity, as increased platelet size (i.e., MPV) has been associated with the presence of low-grade inflammation, and several inflammatory proteins have been proven to influence megakaryocyte maturation and platelet formation [8]. 

Indeed, inflammation is receiving increased attention for its potential role in the pathogenesis of disorders ranging from insulin resistance and type 2 diabetes to fatty liver and CVD [9, 10]. Obesity is associated with a chronic inflammatory response characterized by abnormal adipokine production and the activation of several proinflammatory signalling pathways, resulting in the induction of several biological markers of inflammation [11]. In obese patients, increased accumulation of macrophages is a hallmark of a proinflammatory state that links obesity with systemic inflammation [12]. The foremost physical consequence of obesity is atherosclerosis in CVD [13]. In addition, obesity is accompanied by other clinical complications; these include fatty liver, cholesterol gallstones, sleep apnea, osteoarthritis, and polycystic ovary disease [14].

Adipose tissue has long been considered a nonfunctional storage pool of energy without any direct impact on organ function [15]. However, it has recently been shown that adipose tissue is a secretory organ and a potent source of hormones, peptides, and cytokines involved in food intake regulation, glucose and lipid metabolism, inflammation, coagulation, and blood pressure control [16]. 

 Moreover, it has also become an appealing stem cell source for cell therapy and tissue engineering [17]. Therefore, adipose tissue is now considered to be an active endocrine organ that secretes various humoral factors (adipokines) [18], capable of enhancing the release and production of proinflammatory cytokines in obesity, primarily through nonfat cells, likely contributing to the low-grade systemic inflammatory state found in MS-associated chronic pathologies (e.g., atherosclerosis) [19]. For instance, adiponectin is highly expressed in adipose tissue, and circulating adiponectin levels are decreased in patients with obesity, insulin resistance related to type 2 diabetes, and coronary heart disease [20]. On the other hand, the changes presented by adipose tissue in the setting of MS favor the secretion of several molecular mediators capable of activating or suppressing a number of transcription factors (PPARs, Peroxisome Proliferator Activated Receptors; C/EBPs, CCAAT-enhancer-binding proteins, among other) that regulate different MS-related metabolic pathways [21, 22]. 

The present paper reviews the principal molecular mechanisms involved in adipose tissue inflammation in the setting of MS and provides an in-depth description of the main genetic pathways involved in adipose tissue metabolism.

## 2. Metabolic Syndrome Pathophysiology

The MS is characterized by a high amount of visceral fat, insulin resistance in skeletal muscle, and hypoadiponectinemia [23]. MS subjects showed higher levels of blood pressure, waist circumference, and plasma triglycerides with a high risk of developing type 2 diabetes and CVD in the future [24, 25]. 

The physiopathologic changes associated with MS are diverse including, among others, endothelial dysfunction which triggers atherogenic lesions development and enhanced coagulability [3, 26]. We have reported that MS patients show higher levels of circulating sVCAM-1 and sCD40L, but not sE-selectin, as compared to non-MS patients likely indicating endothelial activation [27, 28]. On the other hand, elevated plasma levels of plasminogen activator inhibitor-1 (PAI-1), tissue factor, and fibrinogen detected in MS patients may contribute to the abnormally increased coagulability further enhancing the risk of CVD [29, 30].

The metabolic changes related to obesity are largely attributable to the amount of intra-abdominal fat mass, rather than total body fat mass [31]. The increased oxidative stress in accumulated fat is an important pathogenic mechanism of obesity-associated MS [32]. The progression of obesity is accompanied by a chronic inflammatory process that involves both innate and acquired immunity [33]. Thus weight loss larger than 10% is associated with an increase in serum adiponectin and a decrease in hs-CRP and plasma fibrinogen [34]. 

The increase of intermuscular adipose tissue was primarily related to age, total body adiposity, and subclinical inflammation [35]. Functional failure of the adipose tissue results in changes in systemic energy delivery, impaired glucose consumption, and activation of self-regulatory mechanisms that extends adipose tissue influence to the whole homeostatic system, by the enhancement of adipokines secretion with the subsequent vascular-related effects [36, 37]. Adipose cell enlargement leads to a cellular proinflammatory state with reduced secretion of adiponectin and increased secretion of IL-6, IL-8, and monocyte chemotactic protein-1 (MCP-1), among others [38]. Data suggest that plasma adiponectin does not change with age, but levels are negatively associated with percent body fat, visceral fat, subcutaneous abdominal fat, insulin, and leptin levels [39, 40]. 

## 3. Adipose Tissue Inflammation in Metabolic Syndrome

There has been a paradigm shift from the traditional notion of adipose tissue merely as an energy storage site to one where adipose tissue plays an active role in energy homeostasis and other various processes [41, 42]. Adipose tissue plays a critical role in energy homeostasis, not only in storing triglycerides, but also responding to nutrient, neural, and hormonal signals and secreting adipokines that regulate feeding, thermogenesis, immunity, and neuroendocrine function [43]. 

Inflammation is increasingly known as a key process underlying metabolic diseases in obese subjects [44]. In particular, adipose tissue-related production of proinflammatory molecules (TNF-*α*, IL-1*β*, IL-6, IL-8, transforming growth factor-*β*, and nerve growth factor) as well as its acute-phase response (plasminogen activator inhibitor-1, haptoglobin, and serum amyloid A) [45] detected during obesity contributes to a low grade of systemic inflammation seen in chronic diseases associated with MS [46–49]. In obese subjects, adiponectin levels are decreased, and the ability of adiponectin to inhibit the inflammatory processes is limited. Low adiponectin levels are inversely related to high levels of C-reactive protein (CRP) in patients with obesity, type 2 diabetes, and CVD [50–52]. In fact, CRP may promote the formation of intimal neovessels in vulnerable atherosclerotic plaques increasing the likelihood of rupture [53].

In mice fed a high-fat diet, weight gain is associated with an induction of adipose tissue-related inflammatory pathways. Thus a high percentage (>50%) of the total adipose tissue mRNA transcripts found to be upregulated during diet-induced weight gain is inflammatory-related genes [47]. In fact, overexpression of low-density lipoprotein receptor-related protein-1 and very low-density lipoprotein receptor in epicardial fat may play a key role in the alterations of lipid metabolism associated with type 2 diabetes mellitus [54]. Thus, 12/15- lipoxygenases (ALOX) and their lipid metabolites, involved in the oxidative metabolism of polyunsaturated fatty acids, act as upstream regulators of many of the cytokines involved in the adipose tissue-related inflammatory response contributing to the development of insulin resistance and diabetes [55]. The gene expression and localization of ALOX isoforms have shown to be exclusively expressed in human visceral fat [56].

Resistin and TNF-*α* are adipokines that have been implicated in insulin resistance in skeletal muscle by the addition of fatty acids to the diacylglycerol [57, 58]. Adipocytes are sensitive to the effects of TNF-*α*, which, through its p55 and p75 TNF receptors, stimulates nuclear factor- (NF-) *κ*B, extracellular signal-regulated kinase, and p38 mitogen-activated protein kinases PI-3 kinase and junN-terminal kinase cascades [59]. The correlation between insulin resistance, chronic inflammation, hypertension, endothelial dysfunction, and dyslipidemia could be due to the activation of NF-*κ*B [60]. The transcription factor NF-*κ*B and the TNF-*α* gene promoter were activated by hypoxia in adipocytes and fibroblasts [61]. NF-*κ*B signaling represses E2F transcription factors eventually inhibiting adipogenesis and maintaining a chronic inflammatory condition [62]. In contrast, hypoxia reduced adiponectin expression, detected d in adipocytes gene promoter [61]. 

TNF-*α* is chronically elevated in adipose tissues of obese rodents and humans. Increased levels of TNF-*α* are implicated in the induction of atherogenic adipokines, such as PAI-1 and IL-6, and the inhibition of the antiatherogenic adipokine, adiponectin [63]. 

Obese individuals have increased TNF-*α* gene expression, as shown by a study in which a 2.5-fold increases in mRNA. Also a strong positive correlation has been detected between TNF-*α* mRNA expression levels and the level of hyperinsulinemia (an indirect measure of insulin resistance) in fat tissue [64].

### 3.1. Role of Adipokines in Chronic Inflammation State: Rheumatoid Arthritis (RA)

Adipokines exert potent modulatory actions on target tissues and cells involved in rheumatic disease [65] and obesity-related diseases [66, 67]. For establishing a relationship with the obesity, using RA as a model, we discuss the participation of adipokines in a chronic inflammation state.

- Adiponectin in RA. A complex adipokine-mediated interaction among white adipose tissue, CVD, and chronic inflammatory autoimmune diseases like RA has been described [68]. In this regard, in RA adipocytes and their surrounding macrophages produce a range of adipokines that regulate systemic inflammation [68]. In RA patients undergoing anti-TNF infliximab therapy because of severe disease, high-grade inflammation shows an independent and negative correlation with circulating adiponectin concentrations, whereas low adiponectin levels clustered with MS features such as dyslipidemia and high plasma glucose levels that have been reportedly to contribute to atherogenesis in RA [69].
- Leptin in RA. In patients undergoing anti-TNF-*α* therapy because of severe disease refractory to conventional therapy, there was a positive correlation between body mass index in patients with RA and leptin serum levels [70]. In addition, these patients showed a significant correlation between leptin levels and VCAM-1 [70, 71]. This is of potential interest as biomarkers of endothelial dysfunction-endothelial cell activation are elevated in patients with RA and anti-TNF blockade improved endothelial dysfunction [72] as well as decreased the levels of endothelial cell activation biomarkers [73].
- Resistin in RA. In patients with RA in treatment with the anti-TNF-*α* monoclonal antibody infliximab for severe disease refractory to conventional therapy, a positive correlation between markers of inflammation, in particular with C-reactive protein, and resistin levels was observed [74]. TNF-*α* blockade led to a rapid reduction in the levels of resistin in these patients [74]. These results highlight a potential role of resistin in the inflammatory cascade in diseases like RA that are associated with chronic inflammatory burden [74].

### 3.2. Leukocytes and Adipose Tissue Inflammation

Macrophage and lymphocyte infiltration in adipose tissue may greatly contribute to obesity-related metabolic dysfunction and chronic inflammation [75, 76]. Recent studies have demonstrated that over 90% of the adipokine release by adipose tissue, except for adiponectin and leptin, could be attributed to nonfat cells [77]. The sequence of events in the inflammatory cascade within the adipose tissue comprises immune cells, first lymphocytes, and then macrophages [78, 79]. 

**Figure 1:**
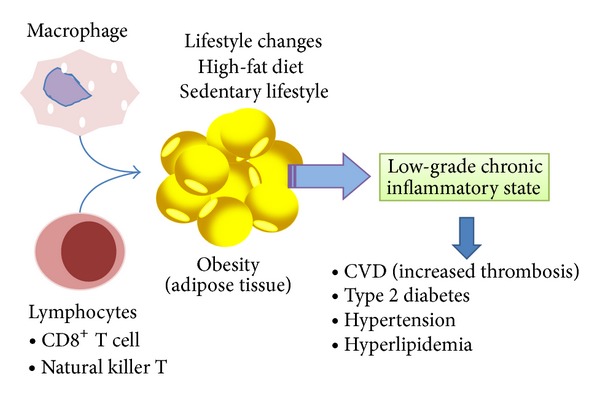
Leukocytes and adipose tissue inflammation. Macrophage and lymphocyte infiltration in adipose tissue may greatly contribute to obesity-related metabolic dysfunction and chronic inflammation. CVD: cardiovascular diseases.

T lymphocytes present in visceral adipose tissue contribute to the initiation and perpetuation of adipose tissue inflammation and the development of insulin resistance [80]. Thus it is observed that a large number of CD8+ effector T cell infiltrate adipose tissue promoting the recruitment and activation of macrophages in obese mice, while the number of CD4+ T cell and regulatory T is diminished (Figure 1) [79]. White adipose tissue hypoxia and CD8+ T cell invasion are features of obesity in C57BL/6J mice and are potential contributors to their local and generalized inflammatory state [81].

Natural killer T (NTK) cells play a crucial role in the development of adipose tissue inflammation and glucose intolerance in diet-induced obesity [82]. However, the deletion of NKT cells, in the absence of alterations in the CD8+ T cell population, is insufficient to protect against the development of the metabolic abnormalities of diet-induced obesity [83].

Macrophage can be characterized as M1-type (involved in proinflammatory processes such as TNF-*α*, IL-6, and IL-12) or immunomodulatory and tissue remodeling (M2). The latter can secrete IL-10, which is an anti-inflammatory cytokine and partake mostly in the downregulation of proinflammatory cytokines [84, 85]. Infiltrated macrophages play the most prominent role, and this low grade inflammation is mediated by the activation and recruitment of macrophages into expanding adipose tissue [12, 86]. In this context, it has been established that the diet-induced obesity leads to a shift in the activation state of adipose tissue macrophages from an M2-type to an M1 proinflammatory state that contributes to insulin resistance [87, 88]. 

### 3.3. Adiponectin as a Regulator of Inflammation

Adiponectin is an abundantly expressed adipokine in adipose tissue and has multiple effects on glucose, metabolism of lipids and free fatty acids, cytokine secretion, and direct insulin sensitizing activity [89, 90]. 

An increase of adiponectin concentrations or the maintenance of higher concentration may be negatively associated with CVD and diabetes, especially in patients with high glycaemic level and independent of adiposity and smoking status [91, 92]. Adiponectin has antiatherosclerotic as well as anti-inflammatory properties that may play an important role in preventing the progression of coronary artery disease [93, 94]. In this context, adiponectin acts on cultured murine and human macrophages to promote a switch to an anti-inflammatory M2 phenotype [95]. Adiponectin can either act directly on macrophages to shift polarization or prime human monocyte differentiation into anti-inflammatory M2 macrophages [95, 96]. Possible pathways of action of adiponectin that leads to a shift in macrophages to an antiinflammatory phenotype include (i) AdipoR1 → IL-10 → HO-1-dependent pathway to decrease TLR4 expression and dampen inflammatory cytokine expression in macrophages and (ii) AdipoR2 → IL-4 → STAT6-dependent signaling pathway that leads to a shift in macrophages to an M2 polarization [97, 98].

Genetic variations in adiponectin receptors (AdipoR1 or AdipoR2) are unlikely to lead to a common genetic predisposition to insulin resistance or type 2 diabetes [99, 100]. Thus, an independent inverse correlation between plasma adiponectin levels and hs-CRP may suggest that decrease of adiponectin contributes to the systemic and vascular inflammation commonly found in obesity [50]. However, patients with advanced heart failure present increased adiponectin with reduced expression of AdipoR1 and AdipoR2 as well as reduced activation of AMP kinase, a known downstream signaling molecule, suggesting a functional adiponectin resistance in advanced heart failure [101]. Also, high levels of adiponectin have been found in chronic inflammatory autoimmune diseases such as SLE, type I diabetes, and rheumatoid arthritis [102–104]. 

Interactions of genetic factors such as single nucleotide polymorphisms (SNPs) in the adiponectin gene and environmental factors causing obesity result in hypoadiponectinaemia, which appears to play an important causal role in obesity-linked insulin resistance, type 2 diabetes, and the MS [105]. In women with MS, visceral fat volume was negatively related to leptin and tended to be negatively related to adiponectin gene expression [106].

 The major genetic modifications of adiponectin are due to oxidative stress generated during obesity. Thus obese subjects exhibit increased systemic oxidative stress, likely derived from the accumulated fat, being an early instigator of MS [32]. A study of 2828 subjects showed that smoking, diabetes, and body mass index were highly associated with systemic oxidative stress and suggest an important role on obesity [107]. Lipid-rich diets are also capable of generating reactive oxygen species because they can alter oxygen metabolism [108]. Increase of free radicals together with low antioxidant capacity detected in obese adults indicate an elevated oxidative stress, which is, in concurrence with systemic inflammation, further potentiated in the case of patients with metabolic syndrome [109].

 An increased oxidative stress is also associated with adiponectin deficiency [110]. Increased oxidative stress has shown to inhibit preadipocyte differentiation as a result of reduced cell proliferation and an inhibition of G(1)→S-phase transition through a transcriptional mechanism involving the inhibition of E2F recruitment and transactivation of its target promoters [111]. Abdominal adiposity and leptin are independent predictors of adiponectin gene expression, and in human adipocytes, adiponectin gene expression is strongly related to I*κ*B-*α* mRNA [112]. The significant independent relationship between adiponectin gene expression and I*κ*B-*α* mRNA suggests that when adiponectin gene expression is high, there is a higher expression of I*κ*B-*α* and the subsequent inhibition of NF-kB transcriptional activity with lower inflammation at the adipocyte level [112].

Adipokine zinc-alpha2-glycoprotein (ZAG) gene expression in adipose tissue is downregulated with increased adiposity and circulating insulin. ZAG mRNA is positively correlated with adiponectin mRNA (ZAG enhances adiponectin production by human adipocytes) [113]. The action of ZAG is associated with downregulated lipogenic enzymes (FAS, ACC1, and DGAT mRNA) and upregulated lipolytic enzyme (HSL mRNA) expressions in adipose tissue [114].

Adiponectin gene transcription is stimulated by several transcription factors involved in adipogenesis such as PPARs, FoxO1, C/EBPs, and SREBPs and is suppressed by hypoxia, inflammation, and transcription repressors such as NFATs and CREB. Proinflammatory cytokines such as TNF-*α*, IL-6, and IL-18 also negatively regulate adiponectin gene transcription by activating several pathways such as the JNK and ERK1/2 pathways [115–117].

## 4. Molecular Interaction and Gene Expression in Adipose Tissue

Changes in the life style, reduction of obesity, and food habits are fundamental in reducing the risk factors [118]. However, there are key factors in MS regulation that depend on those transcription factors that, by responding and adapting to signals from the environment, are able to change the levels of relevant gene expression [119, 120]. Even, gene expression in the metabolic pathways (apoptosis, lipid metabolism, and inflammation) is directly related to the levels of IgM antioxLDL antibodies [121]. Changes in gene expression of adipose tissue suggest that carbohydrate modification can affect the risk of CVD and type 2 diabetes [122]. 

**Figure 2:**
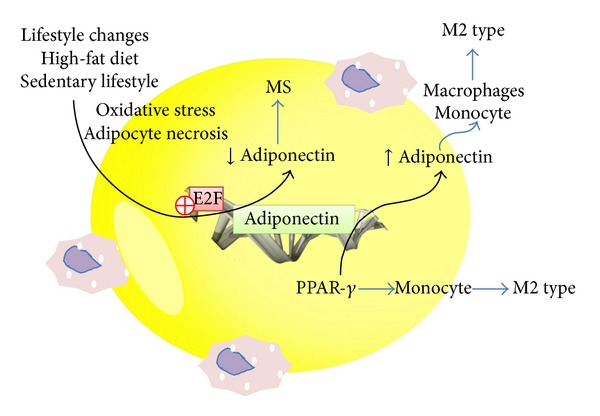
Regulatory pathways of adiponectin in adipose tissue inflammation. M2 type: anti-inflammatory phenotype; MS: metabolic syndrome. ⨁ red circle: inhibition.

The maturation of adipocytes is regulated by a series of transcription factors, mainly PPARs and C/EBPs, that in conjunction regulate the expression of hundreds of proteins that participate in the metabolism and storage of lipids and, as such, the secretion of adipocytes [123]. Meanwhile, the chronic inflammation in adipose tissue is evident from the differential expression of genes involved in inflammatory responses and activation of natural immunity (Figure 2) [124].

*PPARs* are transcriptions factors of a superfamily of nuclear receptors. Three isoforms exist: PPAR-*α*, PPAR-*β* (before PPAR-*δ*), and PPAR-*γ* [125]. The gene expression in the adipose tissue of people with MS seems to be affected by changes in tissue morphology or insulin sensitivity, where a diet high in saturated fatty acids produces a proinflammatory state via the repression of PPARs [126]. The double action of PPAR-*α* and PPAR-*γ* increases the action of adiponectin and the expression of its receptors, which results in an improvement in obesity and a reduction of the inflammatory process [127].

Adipocytes are a major cell target for PPAR-*γ* agonists. This class of compounds includes two drugs, pioglitazone and rosiglitazone, that are widely used to treat patients with diabetes [128]. PPAR-*γ* plays a fundamental role in adipogenesis, as a key regulator in the differentiation and function of adipocytes and the absorption of stored fatty acids [129–131]. 

Meanwhile, it has been suggested recently that PPAR-*γ* is also involved as a key regulator of inflammatory and immune response [132]. PPAR-*γ* is required for maturation of alternatively activated macrophages whatever has a beneficial role in regulating nutrient homeostasis and suggests that macrophage polarization towards the alternative state might be a useful strategy for treating type 2 diabetes [133]. However, PPAR-*γ* activation does not influence M2 marker expression in resting or M1 macrophages, indicating that only native monocytes can be primed by PPAR-*γ* activation to an enhanced M2 phenotype [134]. In addition, PPAR-*γ* transcriptional signaling is required for maturation of an anti-inflammatory M2 phenotype, whereas PPAR-*δ* controls the expression of alternative phenotype in Kupffer cells of obese mice [135].

ApoE expression in adipocytes is regulated by factors involved in modulating systemic insulin sensitivity [136]. Increased plasma apoE levels have been shown to reduce systemic markers of oxidant stress [137]. Adipocytes synthesize and secrete apoE, and its regulation by PPAR-*γ* agonists and TNF-*α* raises an issue regarding the potential significance of adipocyte-derived apoE [138]. TNF-*α* suppresses apoE gene expression in adipocytes, and PPAR-*γ* agonist increases expression of apoE in adipose tissue. Thus TNF-*α* and PPAR-*γ* agonists regulate apoE gene response via distinct apoE gene control elements [139]. For PPAR-*γ*, liver receptor X (LXR) is a key pathway for mediating stimulation of the adipocyte apoE gene [140]. While that TNF-*α* repression of adipocyte apoE gene expression required an intact NF-*κ*B binding site at −43 in the apoE promoter [141].

On the other hand, the loss of function of PPAR-*γ* due to dominant mutations brings about a resistance to insulin and the early onset of severe hypertension [142, 143]. Moreover, IL-6 expression in subcutaneous adipose tissue was significantly associated with intermuscular adipose tissue; IL-6 messenger RNA (mRNA) was negatively associated with adiponectin and PPAR-*γ* expression [35]. Also, loss of PPAR-*γ* in immune cells impairs the ability of abscisic acid to improve insulin sensitivity by suppressing MCP-1 expression and macrophage infiltration into white adipose tissue [144]. 

*C/EBPs* are a family of transcription factors. At least six members of this family have been isolated and characterized to date (C/EBP-*α*-C/EBP-*ζ*) [145]. C/EBP family is essential for the regulation of glucose and lipid homeostasis. C/EBPs-*α*, *β*, and *δ* are tissue specific and highly expressed in adipose tissue [146]. 

C/EBPs and nuclear factor-Y (NF-Y) are critical for the regulation of the adiponectin expression in response to nutrients and in the course of adipocyte differentiation [147]. Even, the transcriptional activity of adiponectin gene during adipocyte differentiation is enhanced by the motif in a novel adiponectin enhancer region, via the recruitment of the C/EBPs and sterol regulatory element-binding proteins (SREBPs) [148, 149]. 

The adiponectin promoter was activated by both C/EBP-*α* and C/EBP-*β*, and the fold increase by C/EBP-*β* was larger than that by C/EBP-*α* [147]. C/EBP-*α* accesses the adiponectin promoter through two forkhead box protein O1 (Foxo1) binding sites and acts as a coactivator. Further, SIRT1 increases adiponectin transcription in adipocytes by activating Foxo1 and enhancing Foxo1 and C/EBP-*α* interaction [150]. Thus low expression of SIRT1 and Foxo1 leads to impaired Foxo1-C/EBP-*α* complex formation, which contributes to the diminished adiponectin expression in obesity and type 2 diabetes [150]. Therefore, C/EBP-*α* is a key transcription factor for full activation of human adiponectin gene transcription in mature adipocytes through interaction with response elements in the intronic enhancer [151].

However, common allelic variants in CEBP-*α* and CEBP-*β* could influence abdominal obesity and related metabolic abnormalities associated with type 2 diabetes and CVD [152]. Meanwhile, activation of PI3 K induced proinflammatory gene expression through activating C/EBP-*β* and C/EBP-*δ* but not NF-*κ*B, which may explain the proinflammatory effect of insulin in the insulin-resistant state [153]. In the hyperinsulinaemic state, C/EBP-*β* leads to the upregulation of CCL2, an inflammation-related protein, which may initiate the process of atherosclerosis [154]. Also C/EBP-*β* activated the TNF-*α* gene promoter, confirming its proinflammatory effect [155].

Moreover, adipose tissue GLUT4 regulates the expression of carbohydrate-responsive-element-binding protein (ChREBP; also known as MLXIPL), a transcriptional regulator of lipogenic and glycolytic genes [156]. ChREBP-*β* expression in human adipose tissue predicts insulin sensitivity, indicating that it may be an effective target for treating diabetes [157]. Moreover, upregulation of GLUT4 gene transcription might be directly mediated by SREBP-1c in adipose tissue [158].

## 5. Conclusion

Obesity triggers a chronic inflammatory state that promotes the production of proinflammatory factors contributing to the impairment of the pathogenesis of MS. The participation of leukocytes plays a critical role in the initiation and propagation of adipose tissue inflammation. Most genetic modifications of adiponectin are due to oxidative stress generated during obesity. Adiponectin and PPAR-*γ* can directly act on macrophages to shift polarization or human monocyte differentiation into anti-inflammatory M2 macrophages. Therefore the clarification of inflammatory processes in the adipose tissue during obesity appears to be essential for the understanding of MS. Thus MS requires the development of new therapeutic strategies addressed to alter the main transcription genetic pathways that regulate adipose tissue metabolism.
